# Selective alteration of human value decisions with medial frontal tDCS is predicted by changes in attractor dynamics

**DOI:** 10.1038/srep25160

**Published:** 2016-05-05

**Authors:** D. Hämmerer, J. Bonaiuto, M. Klein-Flügge, M. Bikson, S. Bestmann

**Affiliations:** 1 Institute of Cognitive Neuroscience, London, United Kingdom; 2 Lifespan Psychology, MPI for Human Development, Berlin, Germany; 3 Sobell Department of Motor Neuroscience and Movement Disorders, UCL Institute of Neurology, University College London, London, United Kingdom; 4 Department of Experimental Psychology, Oxford, United Kingdom; 5 Department for Biomedical Engineering, The City University of New York, New York, NY, USA

## Abstract

During value-based decision making, ventromedial prefrontal cortex (vmPFC) is thought to support choices by tracking the expected gain from different outcomes via a competition-based process. Using a computational neurostimulation approach we asked how perturbing this region might alter this competition and resulting value decisions. We simulated a perturbation of neural dynamics in a biophysically informed model of decision-making through *in silico* depolarization at the level of neuronal ensembles. Simulated depolarization increased baseline firing rates of pyramidal neurons, which altered their susceptibility to background noise, and thereby increased choice stochasticity. These behavioural predictions were compared to choice behaviour in healthy participants performing similar value decisions during transcranial direct current stimulation (tDCS), a non-invasive brain stimulation technique. We placed the soma depolarizing electrode over medial frontal PFC. In line with model predictions, this intervention resulted in more random choices. By contrast, no such effect was observed when placing the depolarizing electrode over lateral PFC. Using a causal manipulation of ventromedial and lateral prefrontal function, these results provide support for competition-based choice dynamics in human vmPFC, and introduce computational neurostimulation as a mechanistic assay for neurostimulation studies of cognition.

The ventromedial prefrontal cortex (vmPFC) is thought to play a key role in guiding value-based decision making^1^,^2^,^3^. For example, BOLD (Blood-oxygenation-level depedendent) activity in vmPFC measured with human neuroimaging scales proportionally to the difference in gain that can be expected when deciding between two options with different expected outcomes^2^,^4^,^5^,^6^,^7^,^8^,^9^,^10^,^11^. Mechanistically, value decisions in vmPFC may emerge through competition between neural populations selective for each choice option^12^,^13^,^14^,^15^. Biophysical attractor models simulate such decisions as a competition between populations of excitatory pyramidal cells whose firing rates are driven by the expected values of one of the choice options as well as background noise (cf. Fig. 1B). Recurrent excitation within, and mutual inhibition between these excitatory populations implement an attractor network with multiple stable attractor states, ultimately resulting in a net activity gain of one population that leads to a decision^13^ (cf. Fig. 1C).

Indeed, this class of biophysical models has been shown to predict activity in vmPFC during decision making^7^,^8^,^16^. Moreover, inter-individual differences in the concentration of the neurotransmitters GABA and glutamate measured in vmPFC predict decision performance across participants^17^. Taken together, these findings corroborate the idea that a competitive process involving inhibitory and excitatory neuronal pools underpins decision making in vmPFC. Finally, evidence from vmPFC lesions in non-human primates also indicates a critical role of vmPFC in value-based decision making^18^,^19^,^20^,^21^. However, there is to date no causal demonstration on how interference with the proposed attractor dynamics in vmPFC may alter choice behaviour. Such a demonstration would provide interventional support for biophysical attractor network models as a candidate mechanism for value-based choice, and could provide mechanistic rationales for interventions aimed at improving decision making in man.

Here, we used a computational neurostimulation approach^22^,^23^ to examine how perturbations of the assumed underlying attractor dynamics in vmPFC might affect decision making behaviour. We first tested, *in silico,* how perturbing the competition between neural populations with neurostimulation affects value-based choice behaviour in a biophysical attractor model (BAM). Perturbations in the BAM were realized through alteration of the membrane polarization^23^,^24^, thus emulating the physiological changes elicited in humans through transcranial direct current stimulation (tDCS). Behavioral predictions generated from this simulated neurostimulation intervention were then compared to the behavioural consequences of an analogous experimental manipulation in human subjects performing a value-based decision making task. Here, we elicited analogous large-scale membrane polarization changes by applying tDCS^25^,^26^ over medial frontal cortex.

A simple approximation of the acute effects of tDCS posits that anodal currents lead to a large-scale net depolarization of pyramidal neurons’ soma within a region, and cathodal currents to a net soma hyperpolarization^27^, thereby influencing neuronal firing rates^27^,^28^,^29^. Commonly, the impact of tDCS on cognitive processing is conceptualized as a sliding-scale of excitability (e.g. anodal stimulation produces “better” processing). It has been suggested that such conceptual models make overly simplistic leaps across several levels of brain organization and do not consider how neural circuits might produce the observed behavioural change^30^,^31^. We therefore have previously advocated simulation of the neural effects of noninvasive brain stimulation in computational models that can generate behavioural responses as a way to address this shortcoming^22^. Here, we used computational neurostimulation to consider how polarization interacts with specific ongoing activity in a decision making attractor, model as well as the resulting choice behaviour. Even though entire populations of neurons in a region will be polarized by tDCS, in this approach the behavioural consequences of stimulation can be linked to the impact on the emerging dynamics of the decision network.

We reasoned that a perturbation of the attractor dynamics would alter the ability to decide between two options of differing value. In cognitive models of decision making, the ability to base decisions on the difference in expected values in the context of background noise is typically captured by the *inverse softmax temperature* parameter, *β*. This parameter reflects how consistently participants choose the subjectively more valuable option, and we will therefore refer to it as the *choice stochasticity*. In other words, a lower *inverse temperature* indicates a larger choice stochasticity, and thus that the option with higher expected value should be chosen less often, which in turn leads to reduced *choice accuracy*. By contrast, a higher *inverse temperature* indicates a lower choice stochasticity, and thus that choices are more driven by value in situations where the difference in expected values is small compared to background noise. In these cases, choice accuracy will increase.

With regard to the BAM, higher choice stochasticity would therefore indicate that attractor dynamics could be more strongly driven by background noise and less strongly driven by the expected values of the choice options. If our manipulation of the attractor dynamics through non-invasive brain stimulation was indeed successful, we would thus expect a comparable change in the model predictions created *in silico* as in our behavioural stimulation study.

## Results

First, we simulated value-based decision making in a biophysical attractor model (BAM) known to describe features of cortical dynamics in decision making through interactions between populations of spiking neurons^12^,^13^. Choices in this network model emerged based on inputs to each pyramidal population that were generated from simulated values of choice options (see Methods). We then simulated changes in neural membrane polarization across the network and their resulting changes on attractor dynamics and choice outcomes. This was done to mirror the physiological changes elicited by tDCS in healthy participants performing value-based decisions, and to allow comparison of model predictions of choice frequencies during stimulation with our empirical human data (see Methods below for details).

Several observations support the choice of the parameters used to simulate polarization of pyramidal cells and inhibitory interneurons during tDCS stimulation. While it is reasonable to assume that large pyramidal cells may exhibit the strongest polarization effects, other neurons are also likely to be affected by polarizing currents^32^,^33^. Our parameter choice was based on prior simulations, where neural network modelling was used to reproduce stimulation-induced changes in sensory evoked potentials *in vitro*, and which suggested that considering the impact of polarizing currents on inhibitory interneuron populations is important^24^. Furthermore, the impact of polarizing currents on membrane potentials in our model closely resembles previous observations in *in-vitro* and *in-vivo* animal experiments^24^,^28^, justifying our choice of stimulation magnitude. In our model, we used minimal assumptions with regards to the impact of polarizing currents, by simply adding transmembrane currents.

### Simulated neurostimulation effects on attractor dynamics and model choice behaviour

To determine the baseline effects of stimulation on the BAM, we inspected the mean firing rate of pyramidal cells and interneurons in the period prior to the onset of the choice stimulus (Fig. 2). Simulated tDCS shifted the mean firing rate of both pyramidal cells (*X*^2^(2) = 194.74, *p* < 0.01) and interneurons (*X*^2^(2) = 190.91, *p* < 0.01). Pairwise comparisons revealed that simulated depolarizing stimulation increased the mean baseline firing rate of pyramidal cells (from 5.81 Hz to 7.71 Hz), but also that of interneurons (0.75 Hz to 1.21 Hz). This pattern arises from an emergent property of the BAM, whereby the increased excitatory drive caused by depolarizing currents to the pyramidal populations increases interneuron population activity, even though the simulated currents for these were hyperpolarizing^24^. Similarly, simulated hyperpolarizing stimulation (which hyperpolarized the pyramidal populations and depolarized the interneuron population) decreased firing rates in all populations (to 4.14 Hz for pyramidal cells and, 0.39 Hz for interneurons; all changes in firing rates Z > 3.5, *p* < 0.01, *r *> 0.70). Relative to no stimulation, the injected current altered the resting membrane potential of each neuron by an amount compatible with *in vitro* studies^32^,^33^,^34^,^35^. The corresponding change in membrane potential varied from −0.2 mV with −4 pA injected current to 0.2 mV with 4 pA injected current^27^,^34^,^35^. There is uncertainty regarding the precise neural elements targeted by tDCS, and our control simulations that omitted direct stimulation of inhibitory interneurons revealed qualitatively similar results. This can be explained by the driving effect the pyramidal populations exert on inhibitory interneurons (see Fig. 3, Supplementary Table 2).

**Figure 1:**
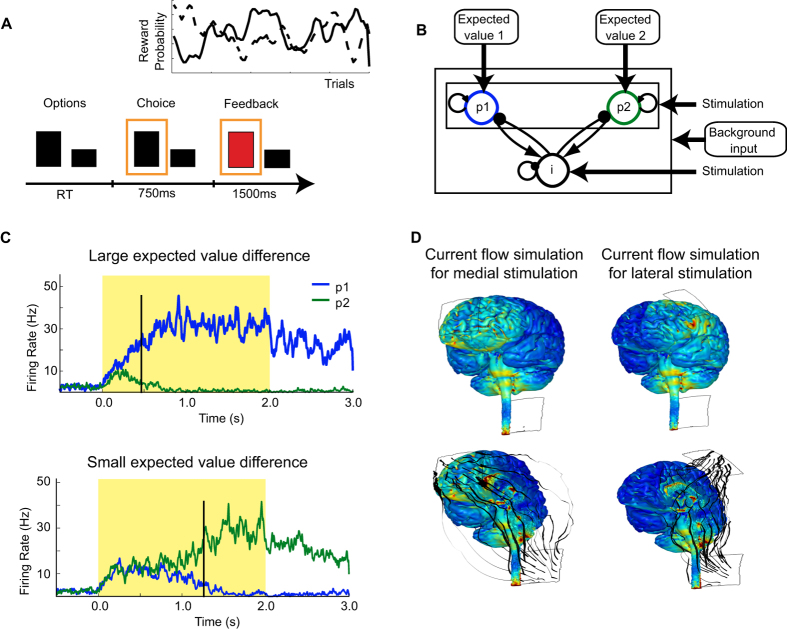
Experimental task, biophysical model and current distribution for medial frontal tDCS. (**A**) Trial structure. Subjects chose between two options of differing reward size (signaled using the size of the rectangle) and probability (learnt). The inset shows one of the random walks used for the reward probabilities of the two options. At the time of choice, the chosen option was indicated (orange rectangle) and the outcome displayed (red: no reward, green: reward) (cf. 5). (**B**) Biophysical model. Excitatory inputs to the two pyramidal populations (*p1* and *p2*, 800 neurons each) are scaled by the expected values of each of the two choice options. Pyramidal populations inhibit each other via a common pool of inhibitory neurons (i, 400 neurons). Background input simulates random firing from regions uninvolved in the task. The effects of tDCS were simulated by changing the membrane potentials of both inhibitory and pyramidal populations (see Methods). (**C**) Temporal development of firing rates of pyramidal populations *p1* and *p2* in the biophysical model. Large expected value differences (upper panel) cause firing rates in the pyramidal populations to differentiate the two inputs earlier, compared to small differences in expected value (lower panel). Vertical black lines: time of predicted response (upon crossing of given threshold, see text for details). Shaded area: onset and duration of choice stimuli and thus the input of expected values. (**D**) Estimated current distributions during medial frontal stimulation (left) and lateral frontal stimulation (right, see Supplementary Fig. 2). Electrode positions are indicated by rectangular outlines (anode over Fpz for medial and over F3 for lateral stimulation condition, cathode below inion in both conditions). Upper row shows lateral view, lower row shows medial view, black lines indicate estimated current flow.

Choice behaviour in the model emerges through the recurrent excitation within pyramidal populations and mutual inhibition between these excitatory populations via an inhibitory interneuron population. A net activity gain of one pyramidal population indicated a decision for one option^13^ (cf. Fig. 1C). To predict qualitative changes in behaviour due to simulated neurostimulation, a standard reinforcement learning (RL) model (see Methods for details) was fitted to the choice predictions generated by the BAM. In evaluating changes in choice behaviour, we examined simulated changes in choice stochasticity, as indicated by the inverse softmax parameter. This analysis therefore asked to what extent choices were driven by noise in neuronal populations versus the relative expected value. In addition, we assessed possible changes in the *learning rate,* indicating to what extent prior decision outcomes influenced current choices. As evident in Fig. 4A (left column), the behavioural model predictions show that depolarizing stimulation increased choice stochasticity (average change *β* of 5.16; *W* = 0.0, *Z* = 3.76, *p* < 0.01, *r* = 0.75). This was also apparent in a decrease in choice accuracy (*X*^2^(2) = 122.92, *p* < 0.01), from 81.28% to 70.24% mean choice accuracy (pairwise-comparison; *Z* = 3.69, *p* < 0.01, *r* = 0.74). The opposite pattern was observed with hyperpolarizing stimulation, which decreased choice stochasticity (average *β* increase of 6.22; *W* = 0.0, *Z* = 3.76, *p* < 0.01, *r* = 0.75) and slightly improved accuracy to 83.95%, (*W* = 57.0, *Z* = 1.86, *p *= 0.06, *r* = 0.37, cf. Fig. 4), although this effect was quantitatively smaller. At the same time, simulated stimulation had no effect on the estimated learning rate (*X*^2^(2) = 2.18, *p* = 0.98; Fig. 4B, left column). In summary, our simulated tDCS intervention thus affected the ability to base decisions on the relative expected value.

**Figure 2:**
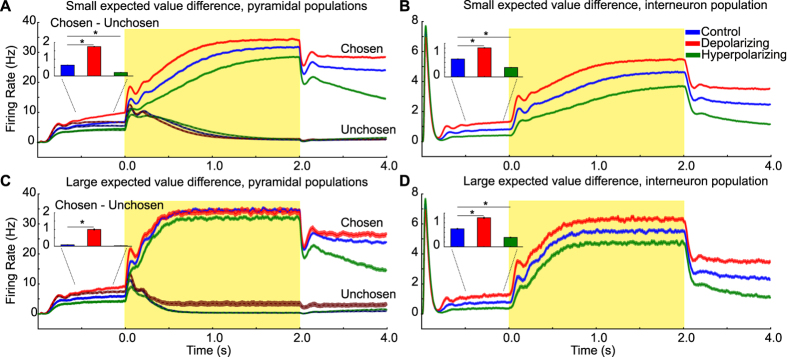
Simulated effects of neurostimulation on mean firing rates of excitatory and inhibitory neuronal populations. (**A**) Mean pyramidal firing rates during trials with small expected value difference. Yellow box indicates period of stimulus-based inputs. Depolarization (red) increases firing rates of pyramidal populations (*p1, p2*), whereas hyperpolarization (green) decreases their firing rates, relative to no stimulation control (blue). Inset shows differences during the pre-stimulus interval (500 to 50 ms before stimulus onset) between the pyramidal populations that reflect the chosen and unchosen option. (**B**) Mean interneuron firing rates during trials with small expected value difference. Depolarization of the network also increases firing rates of inhibitory populations, due to stronger excitatory inputs from pyramidal neurons (cf. Model in Fig. 1), whereas hyperpolarization decreases firing rates. Inset indicates stimulation effects during the pre-stimulus interval, thus paralleling the stimulation effects during stimulus presentation. (**C**) Mean pyramidal firing rates during trials with large differences in expected value. Stimulation (depolarization: red, hyperpolarization: green) has no effect on the steady-state firing rate during visual stimulation relative to control (blue). Inset shows differences during the pre-stimulus interval as in (**A**). Differences in pre-stimulus firing rates between pyramidal populations of chosen and unchosen options are reduced compared to trials with small expected value difference (**A**). Depolarization increases these differences relative to control. (**D**) Mean interneuron firing rates during trials with large differences in expected value. Effects are the same as those during trials with small expected value difference (**B**). Error bars in insets indicate 1 SEM, asterisks indicate reliable condition differences at p < 0.05 (Bonferroni corrected).

A closer look at the temporal dynamics of the network firing rates provides a putative explanation for these qualitative differences in model choice behaviour caused by simulated neurostimulation. The impact of background noise and expected values on choices were differentially affected by neurostimulation. One property of the model used here is that prior to the onset of the choice stimulus, the firing rates of the two pyramidal populations often differ, with one being higher than the other, due to random fluctuations of noisy inputs into the system. Under normal (control) conditions, this asymmetry results in a bias to select the option corresponding to the population with the initially higher firing rate. This effect is stronger when the two choice options have more similar expected values (difficult trials). Crucially, stimulation influences this bias (*X*^2^(2) = 173.31, *p* < 0.01): depolarizing stimulation further amplified this bias (from 0.56 Hz to 1.67 Hz; *W* = 0.0, *Z* = 3.76, *p* < 0.01, *r* = 0.75), whereas hyperpolarizing stimulation reduced it (from 0.56 Hz to 0.18 Hz; *W* = 5.0, *Z* = 3.60, *p* < 0.01, *r* = 0.72; cf Fig. 2). Note that, when examined as choice accuracy on subsets of easy and hard choices, a somewhat more complicated pattern emerges, possibly due to the fact that a modulation of choice accuracy is dependent on overall performance levels (cf. Supplementary Fig. 4).

We next applied a logistic regression to examine the relative influence of pre-stimulus bias and expected value differences on simulated choice behaviour, respectively. As can be seen in Fig. 5, although the network was only provided with the expected value of each response option, expected value differences are a better predictor than pre-stimulus bias for choices under control conditions as indicated by larger beta coefficients (*t*(24) = 4.35, *p* < 0.05, *r* = 0.66). However, simulated depolarizing stimulation increases the relative influence of pre-stimulus bias on choices and thereby increases choice stochasticity (*t*(24) = −5.59, *p* < 0.05, *r* = 0.75). In contrast, hyperpolarizing stimulation reduces the impact of pre-stimulus bias on choice behaviour and thereby decreases choice stochasticity (*t*(24) = 8.27, *p* < 0.05, *r* = 0.86, interaction stimulation condition and bias versus EV influence *F*(2, 48) = 80.83, *p* < 0.05, *r* = 0.87).

### Alternative simulations

**Figure 3:**
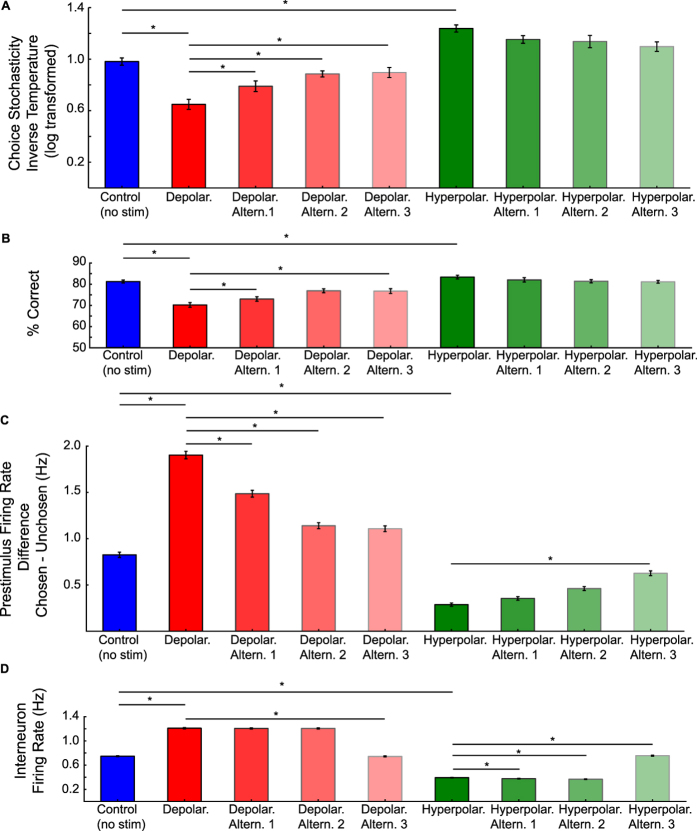
Overview of effects of alternative current simulations. Simulated depolarizing currents (red colours) significantly increased choice stochasiticity (i.e., increased *inverse temperature*). (**A**) and % correct choices (**B**). Furthermore, they increased the difference between the pre-stimulus firing rates of the pyramidal populations (**C**) as well as in interneurons (**D**). Alternative simulations (Altern. 1–2) varying the impact on inhibitory interneurons revealed that these observations were relatively robust to changes in membrane potentials of interneurons. By contrast, no comparable physiological or behavioural change was observed when omitting current to the pyramidal neurons (Depolar. Altern. 3), as expected given current knowledge on how polarizing currents affect pyramidal neurons. Simulated hyperpolarizing currents (green colours) slightly decreased choice stochasticity (i.e., increased *inverse temperature*) (**A**) and thus increased choice accuracy (**B**), and reduced the difference between the pre-stimulus firing rates of the pyramidal populations (**C**) as well as in interneurons (**D**). Similar to depolarizing currents, for hyperpolarizing currents these effects were relatively robust with regards to the specific changes in membrane potentials applied to inhibitory interneurons (Altern. 1–2), but became indistinguishable from baseline (control; blue) simulations when omitting current to the pyramidal neurons (Hyperpolar. Altern. 3). Error bars indicate 1 SEM, asterisks indicate reliable condition differences at p < 0.05 (Bonferroni corrected).

In order to determine whether our modelling results were specific to the parameter values adopted from previous work^24^, we additionally conducted a series of alternative simulations. In doing so, we also simulated the effect of hyperpolarization (cathodal stimulation) on the neural and behavioural output of the biophysical attractor model. Current understanding of the effects of tDCS predicts that hyperpolarizing stimulation ought to cause qualitatively opposite effects on pyramidal neurons; these simulations thus provided a sanity check for the specificity of our modelling results. As for depolarization, estimated learning rates remained unaffected (cf. Fig. 3, Supplementary Table 2) (*W* = 131.0, *Z* = −∞, *p* = 1.0), while hyperpolarizing stimulation decreased choice stochasticity (average *β*: 6.22; *W* = 0.0, *Z* = 3.76, *p* < 0.01, *r* = 0.75, cf. Fig. 3, Supplementary Table 2).

**Figure 4:**
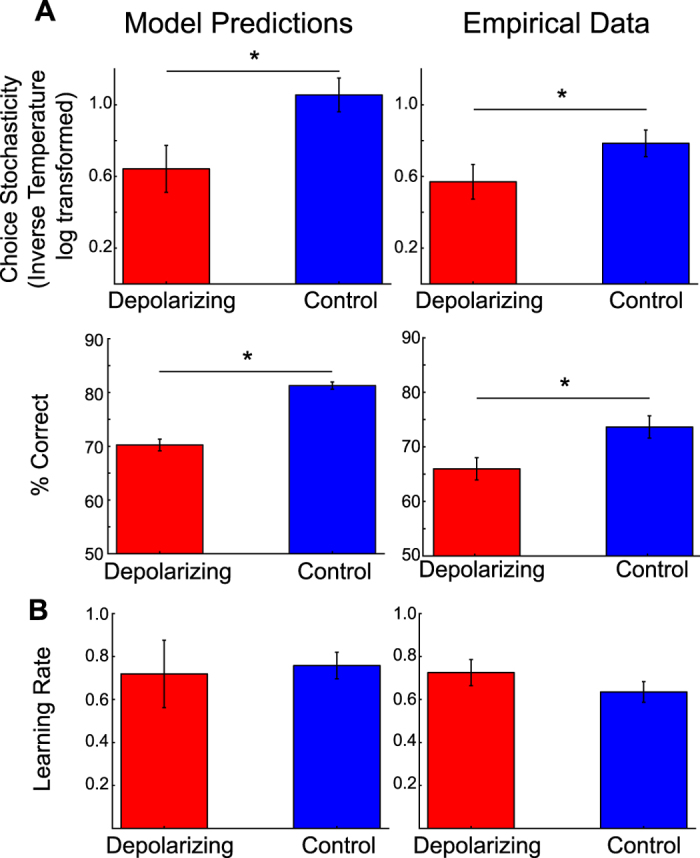
Simulated and empirical effects of neurostimulation on behaviour. (**A**) In line with model predictions (left panel), medial frontal stimulation (depolarization) made decisions less sensitive to differences in expected value between choice options, i.e. increased choice stochasticity. In line with the modeled behaviour (left), mean % correct choices (based on higher expected values calculated as reward magnitude * modelled reward probability) was reduced during medial stimulation (depolarization, right). (**B**) Learning rates were not affected. Error bars indicate 1 SEM, asterisks indicate reliable condition differences at p < 0.05.

**Figure 5:**
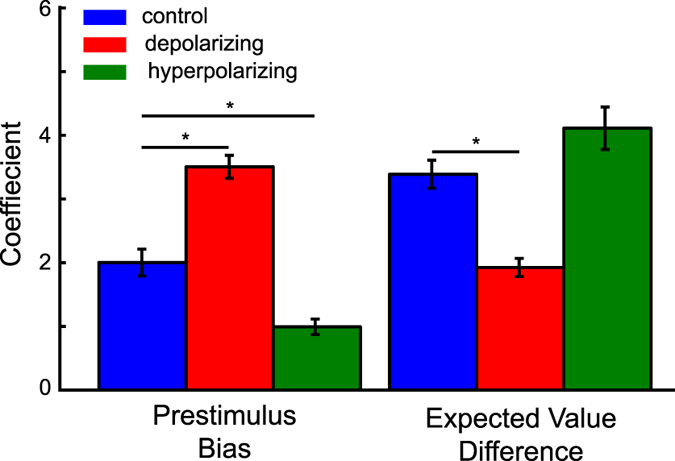
Logistic regression coefficients of pre-stimulus bias and expected value differences predicting accuracy of simulated choices. In the depolarizing stimulation condition, the pre-stimulus pyramidal firing rate bias (correct-incorrect) had a greater influence on simulated choice accuracy than in the control condition, while during hyperpolarizing stimulation the influence of pre-stimulus bias on choice accuracy was reduced compared to control. In contrast, the influence of the expected value difference on choice accuracy was reduced in the depolarizing condition compared to control whereas it didn’t differ reliably in the hyperpolarizing stimulation condition. Thus, hyperpolarization increased the relative bearing of expected value differences on choice accuracy as compared to no stimulation control. In contrast, depolarization increased the relative influence of noisy pre-stimulus fluctuations on choice. Error bars indicate 1 SEM, asterisks indicate reliable condition differences at p < 0.05 (Bonferroni corrected).

We additionally assessed to what degree the results obtained from our main simulations were dependent on the specific parameter values for depolarization and hyperpolarization applied to excitatory and inhibitory populations (cf. Fig. 3, Supplementary Table 2). In our main simulations (cf. Figs 2,4 and 5), we simulated depolarization by injecting 4 pA of current into pyramidal cells and −2 pA into interneurons. As shown in Fig. 3A–D, the overall impact of polarization on choice stochasticity, pre-stimulus bias, percentage correct and firing rates of inhibitory interneurons was relatively robust to the specific current parameters applied. This is because pyramidal populations drive activity in these inhibitory populations, such that an increased firing in the pyramidal population during depolarization would also lead to an increased drive in the inhibitory population^23^. However, the effects of polarization were quantitatively strongest for the parameters adopted in our main simulation, which in turn were based on previous work^24^. By contrast, when applying parameters for stimulation that are in contrast to the known effects of polarizing currents, the impact on the physiological and behavioural model outputs changed significantly (Fig. 3). In these cases, we omitted polarizing current to the pyramidal neurons, which is in stark contrast to known physiology^27^,^28^,^33^. Under these conditions, the effects on behavioural and physiological model predictions became almost indistinguishable from simulations in which no currents were simulated (Fig. 3, Supplementary Table 2), suggesting that the type of simulations conducted here are robust against variations in the specific values chosen for stimulation, but become fragile when stimulation parameters violate basic knowledge about the physiology of tDCS.

### Neurostimulation effects on choice behaviour during value-based decisions in humans

In our behavioural tests, as in our simulated data, choice accuracy during the value-based decision making task was assessed as the percentage of trials in which the option with the higher modelled expected value was chosen (expected value = presented magnitude x modelled probability). Mirroring the predictions from the simulated data, choice accuracy was lower under depolarizing stimulation over vmPFC, as compared to no stimulation (mean difference = −8%, *p* < 0.05, *r* = 0.65; cf. Fig. 4A, right column). By contrast, during the control lateral prefrontal stimulation condition, no reduction in choice accuracy was observed, compared to no stimulation (mean difference = −1%, *p* = 1), and accuracy levels were higher than during medial stimulation (mean difference = 6%, *p* < 0.05, *r* = 0.65) (cf. Supplementary Fig. 2).

Finally, and again in line with the model predictions, choice stochasticity was higher during medial as compared to no stimulation (*Z* = −2.22, *p* < 0.05, *r* = 0.56; Fig. 4A, right column) and compared to lateral prefrontal control stimulation (*Z* = −2.59, *p* < 0.05, *r* = 0.65; cf. Fig. 1 and Supplementary Fig. 2). Lateral prefrontal control stimulation and no stimulation were statistically indistinguishable (*Z* = −1.55, *p* = 0.12). Learning rates were slightly larger during lateral stimulation versus no stimulation (mean lateral stimulation: 0.74, mean medial stimulation: 0.66, mean no stimulation: 0.63; *Z* = 2.0, *p* < 0.05, *r* = 0.50). Moreover, no difference in learning rates was observed between medial and no stimulation (*Z* = 0.6, *p* = 0.50) or medial and lateral stimulation (*Z* = −0.8, *p* = 0.40; Fig. 4B right column, and Supplementary Fig. 2).

The observed effects on choice accuracy were therefore montage specific, causing a decrease in choice performance only during medial stimulation (curvilinear contrast: lateral – medial – no stimulation *F*(1,15) = 15.48, *p* < 0.05, *r* = 0.71). This was also confirmed in pairwise comparisons of the effect of stimulation condition on the percentages of choices of the option with higher subjective expected value (lateral versus medial stimulation: mean difference = 6%, *p* < 0.05, *r* = 0.65; lateral versus no stimulation: mean difference = −1%, *p* = 1). The same was true for RL model parameters as a measure of performance. A comparison of choice stochasticity in the three conditions also revealed a trend for a reliable difference between the three stimulation conditions (Friedman test; *X*^*2*^(2) = 4.88, *p* = 0.09). At the same time, no reliable stimulation effect across the three stimulation conditions was observed for the learning rate parameter (cf. Supplementary Fig. 2, Friedman test; *X*^*2*^(2) = 0.13, *p* = 0.94). Indeed, the learning rate was slightly higher during lateral stimulation (*p* = 0.04, *Z* = 2.0, *r* = 0.50) (cf. Supplementary Fig. 2).

Paralleling the model predictions, we thus found that tDCS stimulation - which is thought to have net soma depolarizing consequences on large populations of pyramidal neurons^23^ - reduced participants’ ability to identify the option with the highest expected value. In the context of the RL model, this stimulation effect was reflected in increased choice stochasticity, consistent with the BAM predictions. A comparison to a control stimulation site over lateral PFC furthermore demonstrated that this effect was montage-specific.

## Discussion

Here we show that neurostimulation over medial frontal cortex can bias value-based decision making, in line with the proposed role for vmPFC. Specifically, our results show that perturbations of the competitive dynamics of a biophysical attractor network model through widespread membrane depolarization impair value-based decision making in a predictable way by modulating the susceptibility of network dynamics to background noise. This resulted in more stochastic simulated choice behaviour. When applying an analogous intervention in healthy participants, we found a similar effect on choice stochasticity. This effect was furthermore spatially specific for medial prefrontal as compared to lateral prefrontal stimulation montages, which provides interventional evidence that is compatible with the idea that competition between neural populations in vmPFC is crucial for value-based choice^7^,^8^,^13^.

The novel *computational neurostimulation* approach used here bridges between the known physiological consequences of tDCS as observed at a cellular level in animal recordings, and the behavioural consequences that can be elicited in humans^22^,^23^,^36^. First, it provides mechanistic predictions of how neurostimulation-related changes in complex neural systems ought to cause behavioural changes^37^. Second, in humans, the neural consequences of targeting large populations of neurons with neurostimulation via tDCS cannot be directly assessed. Using biophysical models at a mesoscopic level such as the one employed here thus provides a novel avenue for predicting *in silico* the emergent properties of large scale networks during neurostimulation^22^,^32^. Indeed, in the context of value decisions, we show that network depolarization can amplify existing (stochastic) biases between neural populations (Fig. 2) and thereby lead to more random choice behaviour (Figs 4 and 5), which provides one of the first mechanistic proposals for how tDCS actually alters behaviour. Third, our approach is a conceptual advance beyond the “sliding-scale” rationale adopted in many prior studies on tDCS and cognitive function, which supposes tDCS simply dials-up (anodal) or down (cathodal) the function of a nominal brain target. Instead, we here considered how polarization by tDCS modulates value decisions through changes in the neuronal network dynamics, rather than a simple sliding scale mechanism.

### Alteration of choice behaviour in the BAM of value-based decision making through simulated neurostimulation

We here used an established BAM of decision making^12^,^13^,^14^,^15^ to predict the neural and resulting behavioural consequences of influencing vmPFC function with neurostimulation. In this model, we simulated changes in the interplay of the excitatory and inhibitory spiking neural populations during value decisions that would be expected to occur during neurostimulation with tDCS, under simplified but reasonable assumptions^23^.

In our simulations, we found that depolarizing network stimulation increased the excitability of pyramidal cells, as well as the excitability of inhibitory interneurons, via a stronger drive from the pyramidal populations. As a consequence, pyramidal populations became more susceptible to background noise (cf. Fig. 2). The explanation for this increased susceptibility lies in the strong recurrent connectivity of the pyramidal populations (cf. Fig. 1B). The increase in pyramidal cell excitability increases the rate of input integration due to these recurrent connections which amplifies both the effects of noisy background inputs prior to the onset of the stimulus and the effective strength of stimulus inputs (cf. Fig. 2). These stimulation effects ultimately increase the rate at which the population with the larger pre-stimulus bias integrates stimulus inputs, and in non-speeded value choices such as the ones tested here result in choices that are more prone to being influenced by pre-stimulus noise (see Fig. 5 and Methods).

Behaviourally, this effect resulted in a reduced ability to select the better choice option in particular when the expected values of choice options were similar (cf. Fig. 4). When examined with a standard RL model, we thus observed an increased randomness of choices with respect to expected values, evident in an increased choice stochasticity (i.e., reduced *inverse temperature*) (cf. Fig. 4A). This result provides one possible mechanism of how large-scale excitability shifts influence the dynamics in BAMs, and how transcranial direct current stimulation may corrupt decision-making processes. Depolarizing stimulation increases the susceptibility to noise inputs and thereby corrupts the interaction between competing attractor states.

### Role of vmPFC for value-computations in humans

In our experimental test of the model predictions, we confirmed the model prediction that depolarizing stimulation over medial prefrontal cortex results in an increased randomness of choices. This was evident in reduced choice accuracy and increased choice stochasticity (cf. Fig. 4A). In both the simulations as well as the human participants, depolarizing stimulation affected in particular the ability to choose based on the relative expected value. Furthermore, stimulation over a control site, with the anode placed over lateral prefrontal cortex, did not yield an effect on choice performance (cf. Supplementary Fig. 2). Our findings thereby lend interventional support to previous reports assigning the vmPFC a key role in value-based decision making^2^,^3^,^4^,^5^,^9^,^10^,^11^,^17^,^18^,^19^,^20^,^21^. Specifically, our results support the idea of competing neural populations as the mechanistic process underpinning this role. But rather than merely being observational, interrogating the impact of stimulation in an established biophysical model provides insight into the underlying physiological change during alterations in decision making.

### Computational neurostimulation in value-based decision making

The *computational neurostimulation* approach used here illustrates how biophysical models at an intermediate (mesoscopic) level of description can generate predictions about the impact of stimulation in biologically plausible networks with emergent properties and dynamics^22^,^38^,^39^. In the present study, this allowed the formation of explicit predictions about the impact of neurostimulation on the stochasticity of value-based choices. Moreover, our results further predict that hyperpolarizing stimulation should have an overall opposite effect on choice performance, namely a decrease in choice stochasticity. Again, this effect ought to be mediated by an alteration in the sensitivity to noise inputs (cf. Fig. 5). Previous work has shown that tDCS can elicit non-linear, intensity-dependent effects^23^,^40^. Assessing the impact of different intensities of hyperpolarizing stimulation on value-based choices systematically in future work may allow for a more detailed characterization of the different behavioural consequences of depolarizing and hyperpolarizing network stimulation.”

More generally, our simulation results provide an example for deriving mechanistically informed rationales for improving value-based decision making, as for example in populations that show impaired value-based decision making due to an increased randomness in choice behaviour as, for example, during healthy ageing^41^,^42^.

### Limitations

The approach taken here approximates neurostimulation effects caused by tDCS by assuming quasi uniform polarization of neuronal populations^32^. However, the anatomical complexity of cortical folding affects current flow^43^,^44^,^45^, but it is currently unknown how local anatomy influences polarization in functionally cogent brain regions. Nonetheless, our approach based on functional targeting and aggregate network activity may make computational neurostimulation predictions robust to such variations.

We here focused on the acute effect of neurostimulation because its impact can be reasonably approximated through modelling changes in transmembrane currents^23^. However, tDCS can also elicit lasting physiological and behavioural changes in neuroplasticity^46^,^47^,^48^,^49^; this calls for development of models that bridge between these more complex physiological changes and the resulting behavioural consequences. Our model does not simulate how physiological processes underlying learning may be affected by stimulation. Incorporating such processes, for example by endowing models with spike-timing dependent plasticity, will be an important next step for future studies. Such development will be of relevance to provide mechanistically informed rationales for translational applications of tDCS.

In our simulations, the main effect of stimulation on neural dynamics was a modulation of the pre-stimulus bias, reflecting the susceptibility of the competition process to background noise. The mechanism through which tDCS exerts its effect when a region simply integrates inputs from other areas without the presence of such competitive dynamics may differ from the process described in our experiment. However, it seems unlikely that the effect of network polarization on neural dynamics even in these cases can be sufficiently explained with a simple dial up/down heuristic. The computational neurostimulation approach as described in the present study will be fruitful for future work to identify the possible mechanistic machinery underlying different types of behaviour and neural computations, including larger scale, multi-region models.

Finally, with regards to the anatomical locus of our results, it is known that current flow occurs in brain regions other than those underlying the stimulation electrodes^22^,^43^,^44^,^48^. Our current simulations provide indication that placing the anode over medial frontal PFC induced currents in this region, whereas placing the anode over a control region (DLPFC) a control region in DLPFC spared this target site. The parameters for the current flow (FEM) model used here are the same as previously used in validation^50^,^51^. The conclusions of our study are conservative in only relying on gross features of current flow (comparison between two very different montages), which are not expected to qualitatively change across a normal range of parameters. We note that ‘no stimulation’ conditions can only provide an indication for performance levels expected during lateral PFC control stimulation as subjects were not blinded with regard to whether tDCS was applied or not. Our non-active control condition therefore provides a baseline for performance levels expected during the lateral PFC control stimulation, but did not account for non-specific stimulation effects. The critical comparison, however, is between the two stimulation sites, which adequately controls for non-specific effects.

Our effects are thus spatially specific with regards to the specific stimulation montages used. However, given the particular position of our electrodes and the known functional neuroanatomy of value-based decision making^52^,^53^,^54^ it is conceivable that direct or indirect stimulation effects could also be observed in subcortical structures, but we note that such complexity would not be unique to our study but indeed to any application of tDCS. Our results are however consistent with a direct stimulation effect to medial frontal cortex causing the observed change in value decision making. Future work may draw on recent advances in combined neurostimulation and neuroimaging measures^55^,^56^ together with finessed models of current flow, computational models of the impact of tDCS, and direct neural recordings, to isolate the specific regions affected by different stimulation protocols.

## Conclusions

In summary, our study provides evidence for a specific role of medial prefrontal cortex (vmPFC) in value-based decision making in healthy humans using neurostimulation over medial prefrontal cortex to directly alter the neural dynamics in that region. Unlike previous stimulation studies, we used a computational neurostimulation approach that provided us with both neuronal and behavioural predictions about the impact of stimulation, and thus a mechanistic interpretational framework for the analogous experiments in our human participants. We observed a striking match in behavioural changes under neurostimulation which was specific to an electrode setup that included vmPFC, compatible with existing theories on the role of the vmPFC during value-based decision making^7^,^8^,^13^,^14^. Finally, with this successful example of linking stimulation interventions with biophysical model predictions we hope to introduce a novel framework for studies aiming at an augmentation of decision making behaviour in populations with altered prefrontal functions.

## Materials and Methods

### Experimental task and procedure

The study was approved by the local ethics committee (UCL research ethics committee) and performed in accordance with the declaration of Helsinki, informed consent was obtained from all participants. Sixteen participants (6 females, mean age: 25.6 years, for details see Supplementary Methods) performed a value-based decision making task (cf. Fig. 1A), during either lateral, medial prefrontal, or no stimulation (see below and Fig. 1D for details on stimulation). The order of the stimulation conditions was balanced across participants. In the value-based decision making task, participants chose between two options that differed in their amount and probability of reward. The amount of money that could be won for each option was indicated by the size of a rectangular bar and varied between 1 and 100 points (Fig. 1A)^7^. The probabilities of winning the respective reward amounts varied according to slow random walks (see Supplementary Methods for details of stimulus generation; cf. inlay Fig. 1A).

### Neurostimulation procedure

Neurostimulation via tDCS was applied using a battery-driven stimulator (DC-Stimulator Plus, neuroConn GmbH, Ilmenau, Germany) during value-based decision making. The stimulating electrodes were inserted in a 5 × 5 cm saline-soaked synthetic sponge and positioned on the participant’s head as illustrated in Fig. 1D. For medial frontal stimulation, the anodal electrode was positioned medially over the forehead (at electrode position Fpz). The cathodal electrode was positioned occipitally right below the inion. Stimulation was applied for 15 min at 2 mA. Stimulation was started 5 minutes before the task. As a control stimulation site, we tested participants on the value-based decision making task while the anodal electrode was positioned over the left lateral frontal cortex (electrode position F3, see Fig. 1D)^29^. Electrode positioning over lateral prefrontal cortex therefore bypassed medial frontal cortex. The lateral control stimulation site is commonly used for studies of working-memory related processes^57^,^58^,^59^,^60^ and thus also served as a control for working-memory related processes. The position of the cathodal electrode was the same for medial and lateral frontal stimulation sites. Figure 1D shows the estimated distributions of electric field (EF) strength across the brain for both electrode montages in an exemplary participant (see Supplementary Methods for details on current simulations).

Our current simulations clearly indicate that medial frontal stimulation can elicit current peaks within the vicinity of ventro-medial prefrontal cortex, in the proximity of the stimulation electrode (see Fig. 1D). By contrast, these calculations suggest that no such current peaks were induced in anterior frontal regions when placing the frontal electrode over the lateral prefrontal cortex. We have previously argued that exclusive stimulation of a brain region with tDCS is impossible^22^,^61^ - in that current will always occur under both electrodes as well as in brain regions between electrodes. Our approach, however, provides support for our effects being montage-specific, and having a strong likelihood of indeed affecting the neural dynamics in medial frontal cortex. Our procedure ensured that the control stimulation over lateral frontal cortex spared our region of interest in medial frontal cortex, whilst inducing comparable currents in other brain regions. This highlights the site specificity of the electrode arrangement.

### Reinforcement learning (RL) model

Subjective reward probabilities underlying choice behaviour during value-based decision making were estimated for each participant by fitting separately for each stimulation condition a standard Rescorla-Wagner reinforcement learning (RL) model^62^ to the participants’ choices (concatenated for the two blocks; probability set to [0.5 0.5] at the start of each block) using the following equation (1):


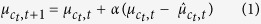


where α is the learning rate which is estimated for each subject, μ_ct_ is the actual reward received in trial *t*, and μ_ct+1_ the expected reward for the chosen stimulus c_t_ in trial t.

Subjective expected values *V*_*1*_ and *V*_*2*_ of the choice options were calculated as the product of modeled probability and offered reward magnitude. The softmax function was used to transform the expected values *V*_*1*_ and *V*_*2*_ of the two options offered on each trial into the probability of choosing option 1.


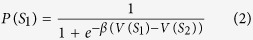


The inverse softmax temperature determines the steepness of the softmax function, and thus how sensitive the choice probability is to differences between the subjective expected values V_1_ and V_2_. For each participant, we thus fitted two parameters (*α (learning rate)*, *β (Inverse temperature)*) using a maximum log-likelihood estimation in Matlab (MathworksMA, USA; version R2014a 8.3.0.532).

### Biophysical attractor model (BAM)

To simulate decision-making behaviour, we used a variation of an established biophysical attractor model (BAM) composed of recurrently connected populations of spiking pyramidal neurons, whose activity reflects the preference for one of the choice options (cf. Fig. 1B)^12^,^13^,^14^. In this model, two populations of pyramidal neurons (*p1* and *p2*, 800 neurons each; Fig. 1B) mutually inhibit each other via a common pool of 400 spiking inhibitory interneurons, and all populations have reciprocal connections with themselves. The pyramidal populations make excitatory synapses (AMPA and NMDA) on target cells and the interneuron population makes inhibitory synapses on its targets. The ratio of excitatory to inhibitory cells was 4:1^52^. Value-related input to pyramidal cells in each excitatory population came in the form of spikes generated from Poisson distributions (see Supplementary Methods and Supplementary Table 1 for details on model parameters).

During stimulus presentation, the firing rate of each task-related input pool, *f*_*i,t*_, was scaled according to the expected value of the associated response option, *i*:


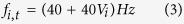


To simulate non-task-related (background) input, each neuron additionally received a common set of spike inputs from a single Poisson distribution at a set rate. Mean population firing rates were computed by convolving the instantaneous firing rate with a Gaussian filter with a width of 5 ms. During simulated value-based decision making, the firing rates of the pyramidal populations converged to a pattern in which differences in the inputs were magnified and ultimately prompted the selection of one response once one of the pyramidal populations exceeded a set response threshold of 25 Hz (cf. Fig. 1C). For similar expected values of the two choice options, each population received roughly the same level of input stimulation and therefore response selection was slower and more random due to similar levels of background noise (cf. Fig. 1B). Previous studies show that this model is well suited for decision-making among choice options^7^,^8^,^63^.

To simulate value-based decision making during the course of the task, the BAM was provided with inputs from the same Rescorla-Wagner rule that was part of the standard RL model used for modelling the behavioural experimental data. Specifically, the expected reward probability of each response option on the current trial was updated using the Rescorla-Wagner rule, and the expected value was computed by multiplying the expected probability by the offered reward magnitude as was done for the modelling of the behavioural data (see above). To analyse the behavioural output of the BAM, its choices were then fitted using the same standard RL model used to fit the behavioural experimental data (including the Rescorla-Wagner rule and softmax function).

The impact of tDCS on the ability to make value decisions was simulated in the BAM by altering the membrane potential parameters for pyramidal cells and inhibitory interneurons, based on values from simulations reproducing tDCS-induced changes in sensory evoked potentials *in vivo*^23^ and current understanding of the mechanism of action of tDCS^25^,^32^,^33^,^34^,^64^,^65^. Specifically, anodal (depolarizing) stimulation was simulated by ‘injecting’ 4 pA of current into each pyramidal cell and −2 pA into each interneuron^23^. Relative to no stimulation, the injected current changed the resting membrane potential of each neuron by a small amount. This change in membrane potential varied from ±0.1 mV with ±2 pA injected current to ±0.2 mV with ±4 pA injected current, which reassuringly is within the range found by *in vitro* studies of the effects of tDCS^25^,^29^,^34^.

When generating behavioural predictions about the effect of neurostimulation in humans, we first ran the BAM using a range of *learning rate* and *inverse temperature* values previously reported during a similar probabilistic value-based decision making task (*learning rate* range: [0.0,1.0], *inverse temperature* range: [1.69, 6.63])^2^. Here the learning rate was used by the Rescorla-Wagner rule used to provide inputs to the BAM and the *inverse temperature* value was used to scale the magnitude of the background input to the BAM (see above). For each learning rate and *inverse temperature* value tested, we ran the model with and without simulated depolarizing stimulation to determine how the stimulation affected the learning rate and *inverse temperature* estimated from the model’s behaviour.

Following stimulation of our participants, we used the behavioural model fits during the no stimulation control condition to create virtual subjects, and thus simulate choice behaviour in the BAM. This allowed for comparison between previously published parameters for learning rates and *inverse temperatures*, and the parameters obtained in our cohort. Specifically, we generated virtual subjects by sampling the distribution of *learning rates* and *inverse temperatures* resulting from behavioural model fits (*learning rate* range: [0.33,0.88], *inverse temperature* range: [0.43, 12.31]). These virtual subjects were then used to form behavioural predictions for the stimulation conditions. Each virtual subject was run using the same choice stimuli as used for the actual subjects in the experimental data. All simulations were implemented in the Python programming language using the Brian simulator^66^.

## Additional Information

**How to cite this article**: Hämmerer, D. *et al.* Selective alteration of human value decisions with medial frontal tDCS is predicted by changes in attractor dynamics. *Sci. Rep.* **6**, 25160; doi: 10.1038/srep25160 (2016).

## Supplementary Material

Supplementary Information 1

## References

[b1] Deco G., Rolls E. T., Albantakis L. & Romo R. Brain mechanisms for perceptual and reward-related decision-making. Prog. Neurobiol. 103, 194–213 (2013). 22326926 10.1016/j.pneurobio.2012.01.010

[b2] O’Doherty J. P. Contributions of the ventromedial prefrontal cortex to goal-directed action selection. Ann. N. Y. Acad. Sci. 1239, 118–129 (2011). 22145881 10.1111/j.1749-6632.2011.06290.x

[b3] Rushworth M. F., Mars R. B. & Summerfield C. General mechanisms for making decisions? Curr. Opin. Neurobiol. 19, 75–83 (2009). 19349160 10.1016/j.conb.2009.02.005

[b4] Basten U., Biele G., Heekeren H. R. & Fiebach C. J. How the brain integrates costs and benefits during decision making. Proc. Natl. Acad. Sci. 107, 21767–21772 (2010). 21118983 10.1073/pnas.0908104107 PMC3003102

[b5] Behrens T. E. J., Woolrich M. W., Walton M. E. & Rushworth M. F. S. Learning the value of information in an uncertain world. Nat. Neurosci. 10, 1214–1221 (2007). 17676057 10.1038/nn1954

[b6] Boorman E. D., Behrens T. E. J., Woolrich M. W. & Rushworth M. F. S. How Green Is the Grass on the Other Side? Frontopolar Cortex and the Evidence in Favor of Alternative Courses of Action. Neuron 62, 733–743 (2009). 19524531 10.1016/j.neuron.2009.05.014

[b7] Hunt L. T. *et al.* Mechanisms underlying cortical activity during value-guided choice. Nat. Neurosci. 15, 470–476 (2012). 22231429 10.1038/nn.3017 PMC3378494

[b8] Hunt L. T., Dolan R. J. & Behrens T. E. J. Hierarchical competitions subserving multi-attribute choice. Nat. Neurosci. 17, 1613–1622 (2014). 25306549 10.1038/nn.3836 PMC4685756

[b9] Kolling N., Behrens T. E. J., Mars R. B. & Rushworth M. F. S. Neural Mechanisms of Foraging. Science 336, 95–98 (2012). 22491854 10.1126/science.1216930 PMC3440844

[b10] Philiastides M. G., Biele G. & Heekeren H. R. A mechanistic account of value computation in the human brain. Proc. Natl. Acad. Sci. 107, 9430–9435 (2010). 20439711 10.1073/pnas.1001732107 PMC2889112

[b11] Wunderlich K., Dayan P. & Dolan R. J. Mapping value based planning and extensively trained choice in the human brain. Nat. Neurosci. 15, 786–791 (2012). 22406551 10.1038/nn.3068 PMC3378641

[b12] Bonaiuto J. & Arbib M. A. Modeling the BOLD correlates of competitive neural dynamics. Neural Netw. Off. J. Int. Neural Netw. Soc. 49, 1–10 (2014). 10.1016/j.neunet.2013.09.001 24076766

[b13] Wang X.-J. Probabilistic decision making by slow reverberation in cortical circuits. Neuron 36, 955–968 (2002). 12467598 10.1016/s0896-6273(02)01092-9

[b14] Wang X.-J. Decision Making in Recurrent Neuronal Circuits. Neuron 60, 215–234 (2008). 18957215 10.1016/j.neuron.2008.09.034 PMC2710297

[b15] Wong K.-F., Huk A. C., Shadlen M. N. & Wang X.-J. Neural circuit dynamics underlying accumulation of time-varying evidence during perceptual decision making. Front. Comput. Neurosci. 1, 6 (2007). 18946528 10.3389/neuro.10.006.2007 PMC2525934

[b16] Hunt L. T. What are the neural origins of choice variability? Trends Cogn. Sci. 18, 222–224 (2014). 24513295 10.1016/j.tics.2014.01.004 PMC4888938

[b17] Jocham G., Hunt L. T., Near J. & Behrens T. E. J. A mechanism for value-guided choice based on the excitation-inhibition balance in prefrontal cortex. Nat. Neurosci. 15, 960–961 (2012). 22706268 10.1038/nn.3140 PMC4050076

[b18] Fellows L. K. & Farah M. J. The Role of Ventromedial Prefrontal Cortex in Decision Making: Judgment under Uncertainty or Judgment Per Se? Cereb. Cortex 17, 2669–2674 (2007). 17259643 10.1093/cercor/bhl176

[b19] Noonan M. P. *et al.* Separate value comparison and learning mechanisms in macaque medial and lateral orbitofrontal cortex. Proc. Natl. Acad. Sci. 107, 20547–20552 (2010). 21059901 10.1073/pnas.1012246107 PMC2996698

[b20] Rudebeck P. H. & Murray E. A. Dissociable effects of subtotal lesions within the macaque orbital prefrontal cortex on reward-guided behaviour. J. Neurosci. Off. J. Soc. Neurosci. 31, 10569–10578 (2011). 10.1523/JNEUROSCI.0091-11.2011 PMC3171204 21775601

[b21] Rudebeck P. H. & Murray E. A. Balkanizing the primate orbitofrontal cortex: distinct subregions for comparing and contrasting values. Ann. N. Y. Acad. Sci. 1239, 1–13 (2011). 22145870 10.1111/j.1749-6632.2011.06267.x PMC3951748

[b22] Bestmann S., de Berker A. O. & Bonaiuto J. Understanding the behavioural consequences of noninvasive brain stimulation. Trends Cogn. Sci. 19, 13–20 (2015). 25467129 10.1016/j.tics.2014.10.003

[b23] Bonaiuto J. & Bestmann S. Understanding the nonlinear physiological and behavioural effects of tDCS through computational neurostimulation. Prog. Brain Res. In press (2015). 10.1016/bs.pbr.2015.06.013 26541377

[b24] Molaee-Ardekani B. *et al.* Effects of transcranial Direct Current Stimulation (tDCS) on cortical activity: A computational modelling study. Brain Stimulat. 6, 25–39 (2013). 10.1016/j.brs.2011.12.006 22420944

[b25] Nitsche & Paulus W. Transcranial direct current stimulation – update 2011. Restor. Neurol. Neurosci. 29, 463–492 (2011). 22085959 10.3233/RNN-2011-0618

[b26] Nitsche *et al.* Transcranial direct current stimulation: State of the art 2008. Brain Stimulat. 1, 206–223 (2008). 10.1016/j.brs.2008.06.004 20633386

[b27] Rahman A. *et al.* Cellular effects of acute direct current stimulation: somatic and synaptic terminal effects. J. Physiol. 591, 2563–2578 (2013). 23478132 10.1113/jphysiol.2012.247171 PMC3678043

[b28] Márquez-Ruiz J. *et al.* Transcranial direct-current stimulation modulates synaptic mechanisms involved in associative learning in behaving rabbits. Proc. Natl. Acad. Sci. 109, 6710–6715 (2012). 22493252 10.1073/pnas.1121147109 PMC3340065

[b29] Nitsche M. A. *et al.* Shaping the Effects of Transcranial Direct Current Stimulation of the Human Motor Cortex. J. Neurophysiol. 97, 3109–3117 (2007). 17251360 10.1152/jn.01312.2006

[b30] Antal A. *et al.* Direct current stimulation over V5 enhances visuomotor coordination by improving motion perception in humans. J. Cogn. Neurosci. 16, 521–527 (2004). 15165345 10.1162/089892904323057263

[b31] Miniussi C., Harris J. A. & Ruzzoli M. Modelling non-invasive brain stimulation in cognitive neuroscience. Neurosci. Biobehav. Rev. 37, 1702–1712 (2013). 23827785 10.1016/j.neubiorev.2013.06.014

[b32] Bikson M. *et al.* Modeling sequence and quasi-uniform assumption in computational neurostimulation. Prog. Brain Res. 222, 1–23, 10.1016/bs.pbr.2015.08.005 (2015) 26541374

[b33] Rahman R. A., Lafon B. & Bikson M. Multilevel Computational Models for Predicting the Cellular Effects of Non-Invasive Brain Stimulation. Prog. Brain Res. In press (2015). 10.1016/bs.pbr.2015.09.003 26541375

[b34] Bikson M. *et al.* Effects of uniform extracellular DC electric fields on excitability in rat hippocampal slices *in vitro*. J. Physiol. 557, 175–190 (2004). 14978199 10.1113/jphysiol.2003.055772 PMC1665051

[b35] Radman T., Ramos R. L., Brumberg J. C. & Bikson M. Role of cortical cell type and morphology in subthreshold and suprathreshold uniform electric field stimulation *in vitro*. Brain Stimulat. 2, 215–228, 228.e1–3 (2009). 10.1016/j.brs.2009.03.007 PMC2797131 20161507

[b36] Bestmann S. & Little. Computational Neurostimulation for Parkinson’s Disease. Prog. Brain Res. in press (2015). 10.1016/bs.pbr.2015.09.002 26541381

[b37] Kutchko K. M. & Fröhlich F. Emergence of Metastable State Dynamics in Interconnected Cortical Networks with Propagation Delays. PLoS Comput Biol 9, e1003304 (2013). 24204238 10.1371/journal.pcbi.1003304 PMC3812055

[b38] Eshel N., Tian J. & Uchida N. Opening the black box: dopamine, predictions, and learning. Trends Cogn. Sci. 17, 430–431 (2013). 23830895 10.1016/j.tics.2013.06.010 PMC3811049

[b39] Maia T. V. & Frank M. J. From reinforcement learning models to psychiatric and neurological disorders. Nat. Neurosci. 14, 154–162 (2011). 21270784 10.1038/nn.2723 PMC4408000

[b40] Batsikadze G., Moliadze V., Paulus W., Kuo M.-F. & Nitsche M. A. Partially non-linear stimulation intensity-dependent effects of direct current stimulation on motor cortex excitability in humans. J. Physiol. 591, 1987–2000 (2013). 23339180 10.1113/jphysiol.2012.249730 PMC3624864

[b41] Hämmerer D. & Eppinger B. Dopaminergic and prefrontal contributions to reward-based learning and outcome monitoring during child development and aging. Dev. Psychol. 48, 862–874 (2012). 22390655 10.1037/a0027342

[b42] Hämmerer D., Li S.-C., Müller V. & Lindenberger U. Life span differences in electrophysiological correlates of monitoring gains and losses during probabilistic reinforcement learning. J. Cogn. Neurosci. 23, 579–592 (2011). 20377358 10.1162/jocn.2010.21475

[b43] Bikson M., Rahman A. & Datta A. Computational models of transcranial direct current stimulation. Clin. EEG Neurosci. 43, 176–183 (2012). 22956646 10.1177/1550059412445138

[b44] Bikson M., Rahman A., Datta A., Fregni F. & Merabet L. High-resolution modelling assisted design of customized and individualized transcranial direct current stimulation protocols. Neuromodulation J. Int. Neuromodulation Soc. 15, 306–315 (2012). 10.1111/j.1525-1403.2012.00481.x PMC3418452 22780230

[b45] Salvador R., Mekonnen A., Ruffini G. & Miranda P. C. Modeling the electric field induced in a high resolution realistic head model during transcranial current stimulation. *Conf. Proc. Annu. Int. Conf. IEEE Eng. Med. Biol. Soc. IEEE Eng. Med. Biol. Soc. Annu. Conf.* 2010, 2073–2076 (2010). 10.1109/IEMBS.2010.5626315 21095946

[b46] Bikson M., Radman T. & Datta A. Rational modulation of neuronal processing with applied electric fields. Conf. Proc. Annu. Int. Conf. IEEE Eng. Med. Biol. Soc. IEEE Eng. Med. Biol. Soc. Annu. Conf. 1, 1616–1619 (2006). 10.1109/IEMBS.2006.259548 17946911

[b47] Fritsch B. *et al.* Direct current stimulation promotes BDNF-dependent synaptic plasticity: potential implications for motor learning. Neuron 66, 198–204 (2010). 20434997 10.1016/j.neuron.2010.03.035 PMC2864780

[b48] Reato D., Bikson M. & Parra L. C. Lasting modulation of *in vitro* oscillatory activity with weak direct current stimulation. J. Neurophysiol. 113, 1334–1341 (2015). 25505103 10.1152/jn.00208.2014 PMC4346723

[b49] Reis J. *et al.* Noninvasive cortical stimulation enhances motor skill acquisition over multiple days through an effect on consolidation. Proc. Natl. Acad. Sci. USA 106, 1590–1595 (2009). 19164589 10.1073/pnas.0805413106 PMC2635787

[b50] Datta A. *et al.* Gyri-precise head model of transcranial direct current stimulation: Improved spatial focality using a ring electrode versus conventional rectangular pad. Brain Stimulat. 2, 201–207, e1 (2009). 10.1016/j.brs.2009.03.005 PMC2790295 20648973

[b51] Edwards D. *et al.* Physiological and modelling evidence for focal transcranial electrical brain stimulation in humans: A basis for high-definition tDCS. NeuroImage 74, 266–275 (2013). 23370061 10.1016/j.neuroimage.2013.01.042 PMC4359173

[b52] Braitenberg V. & Schüz A. Anatomy of the Cortex: Statistics and Geometry (Springer, 1991).

[b53] Fellows L. K. Orbitofrontal contributions to value-based decision making: evidence from humans with frontal lobe damage. Ann. N. Y. Acad. Sci. 1239, 51–58 (2011). 22145875 10.1111/j.1749-6632.2011.06229.x

[b54] Padoa-Schioppa C. Neurobiology of economic choice: a good-based model. Annu. Rev. Neurosci. 34, 333–359 (2011). 21456961 10.1146/annurev-neuro-061010-113648 PMC3273993

[b55] Bestmann S. & Feredoes E. Combined neurostimulation and neuroimaging in cognitive neuroscience: past, present, and future. Ann. N. Y. Acad. Sci. 1296, 11–30 (2013). 23631540 10.1111/nyas.12110 PMC3760762

[b56] Soekadar S. R. *et al.* *In vivo* assessment of human brain oscillations during application of transcranial electric currents. Nat. Commun. 4, 2032 (2013). 23787780 10.1038/ncomms3032 PMC4892116

[b57] Andrews S. C., Hoy K. E., Enticott P. G., Daskalakis Z. J. & Fitzgerald P. B. Improving working memory: the effect of combining cognitive activity and anodal transcranial direct current stimulation to the left dorsolateral prefrontal cortex. Brain Stimulat. 4, 84–89 (2011). 10.1016/j.brs.2010.06.004 21511208

[b58] Fregni F. *et al.* Anodal transcranial direct current stimulation of prefrontal cortex enhances working memory. Exp. Brain Res. 166, 23–30 (2005). 15999258 10.1007/s00221-005-2334-6

[b59] Knoch D., Pascual-Leone A., Meyer K., Treyer V. & Fehr E. Diminishing Reciprocal Fairness by Disrupting the Right Prefrontal Cortex. Science 314, 829–832 (2006). 17023614 10.1126/science.1129156

[b60] Knoch D. *et al.* Studying the Neurobiology of Social Interaction with Transcranial Direct Current Stimulation–The Example of Punishing Unfairness. Cereb. Cortex 18, 1987–1990 (2008). 18158325 10.1093/cercor/bhm237

[b61] De Berker A. O., Bikson M. & Bestmann S. Predicting the behavioural impact of transcranial direct current stimulation: issues and limitations. Front. Hum. Neurosci. 7, 1–6 (2013). 24109445 10.3389/fnhum.2013.00613 PMC3790257

[b62] Rescorla R. A. & Wagner A. R. A theory of Pavlovian conditioning: Variations in the effectiveness of reinforcement and nonreinforcement. In Classical conditioning II: Current research and theory (eds. Black A. H. & Prokasy, W. F.) (1972).

[b63] Usher M. & McClelland J. L. The time course of perceptual choice: The leaky, competing accumulator model. Psychol. Rev. 108, 550–592 (2001). 11488378 10.1037/0033-295x.108.3.550

[b64] Bindman L. J., Lippold O. C. & Redfearn J. W. The action of brief polarizing currents on the cerebral cortex of the rat (1) during current flow and (2) in the production of long-lasting after-effects. J. Physiol. 172, 369–382 (1964). 14199369 10.1113/jphysiol.1964.sp007425 PMC1368854

[b65] Funke K. Quite simple at first glance - complex at a second: modulating neuronal activity by tDCS. J. Physiol. 591, 3809 (2013). 23950162 10.1113/jphysiol.2013.260661 PMC3764629

[b66] Goodman D. & Brette R. Brian: A Simulator for Spiking Neural Networks in Python. Front. Neuroinformatics 2, 1–10 (2008). 10.3389/neuro.11.005.2008 PMC2605403 19115011

