# Supplementary material for: Selective alteration of human value decisions with medial frontal tDCS is predicted by changes in attractor dynamics
Source: Sci Rep. 2016 May 5;6:25160. doi: 10.1038/srep25160 (PMC4857193; doi:10.1038/srep25160)
Supplement: Supplementary Information 1 [file srep25160-s1.pdf]

Selective alteration of human value decisions with medial frontal tDCS is predicted by changes in attractor dynamics

Hämmerer, D[*1,2], Bonaiuto, J. [*3] , Klein-Flügge, M. [*3,4], Bikson, M.[5], Bestmann, S.[3]

* these authors contributed equally

Supplementary Methods

Participants

Twenty-four participants with no history of psychiatric or neurological disorder took part in the study. Consumption of recreational substances or other medications were assessed via self-report. Two participants were excluded because they failed to comply with task instructions. An essential component of the behavioural task was the tracking of reward probability because it ensured that (a) choices were not purely driven by the reward magnitude displayed on the screen, or (b) determined by other strategies that would be at odds with the instructions, such as consistently choosing with one hand, choosing randomly, or a simple 1-back rule. For this reason, six further subjects had to be excluded as their choice behaviour suggested that they did not integrate rewards over the recent history of trials, which is required for estimating the current reward probability. This was evidenced by learning rates <0.05 or >0.95 in the session where no stimulation was applied.

The final sample included 16 participants (6 females, mean age: 25.6 years, range: 20-37 years, std: 5.4 years) who were tested across four experimental sessions. During those repeated test sessions, participants also worked on a perceptual decision making task as part of another experiment. Average task duration for the decision making task was 13.5 min. On average, stimulation therefore terminated about 3 minutes before the task finished. A comparison of choice accuracy of the whole dataset and a dataset excluding the last third of the trials did not show significantly different results (see Supplementary Figure 1 below). We would therefore assume that the termination of the stimulation before task completion did not affect our results to a statistically measurable degree. All participants gave written informed consent and were screened for suitability for non-invasive brain stimulation according to standard procedures. The study was approved by the local ethics committee and performed in accordance with the declaration of Helsinki. Participants received £10 per completed hour of testing, up to an additional £5 depending on performance, and an additional £10 upon completion of all experimental sessions.

Generation of choice stimuli

During the task (see Methods), the probability of winning the reward amounts shown on the screen had to be inferred. Probabilities varied according to random walks generated with the following parameters: smoothed in sliding time window of 5 trials, decay parameter =.80, decay centre =.50, diffusion noise =.08, diffusion mean =0. Random walks and reward magnitudes were chosen such that correlations between probabilities, magnitudes, and expected values within as well as between the two options were minimal (all r^2 below .50, average r^2=.13). Furthermore, magnitudes and probabilities were distributed across choices such that the relative expected values for the two choice options was small on the majority of trials (on 80% of the trials, the relative expected value between the two options was kept below .20). This was done because we were specifically interested in the process of choice computation and hence the processing of relative expected values (the difference in expected value of the two choice

options, i.e., magnitude x probability). By making the relative expected values small, the choice computation process was made particularly hard.

Biophysical Attractor Model (BAM)

The biophysical attractor model (BAM) contains two populations of pyramidal cells, one for each response option, and one population of inhibitory interneurons [1–4]. Each pyramidal population is reciprocally connected with the interneuron population, and all populations have reciprocal connections with themselves. The pyramidal populations make excitatory synapses (AMPA and NMDA) on target cells and the interneuron population makes inhibitory synapses on its targets.

Synapses are modelled as exponential (AMPA, $GABA_A$) or bi-exponential conductances for slower rising synapses (NMDA). Exponentially decaying conductances are governed by the following equation (1):

$$g(t) = Ge^{-t/\tau} \tag{1}$$

where G is the maximal conductance (or weight) of that specific synapse, and τ is a synapse type-specific decay time constant. Similarly, bi-exponential synaptic conductances were given by:

$$g(t) = G\frac{\tau_2}{\tau_2 - \tau_1}\left(e^{-t/\tau_1} - e^{-t/\tau_2}\right) \tag{2}$$

where τ_1 and τ_2 are synapse type-specific decay and rise time constants (see Appendix for parameter values for each synapse type). Synaptic currents were generated using these conductances and the associated reversal potential, E:

$$I(t) = g(t)\left(V_m - E\right) \tag{3}$$

where V_m is the membrane voltage. The additional voltage dependence of NMDA synapses was captured by:

$$I_{NMDA}(t) = \frac{g_{NMDA}(t)\left(V_m - E_{NMDA}\right)}{1 + \left[Mg^{2+}\right]\exp\left(-0.062V_m\right)/3.57} \tag{4}$$

where $[Mg^{2+}]$ is the extracellular magnesium concentration.

The synaptic currents of each type (AMPA, NMDA, and $GABA_A$) were summed and the total synaptic current was input into the adaptive exponential leaky integrate-and-fire (LIF) neural model (Brette and Gerstner, 2005)

$$I_{total}(t) = I_{AMPA}(t) + I_{NMDA}(t) + I_{GABA_A}(t) \tag{5}$$

$$C\frac{dV_m}{dt} = g_L\left(V_m - E_L\right) + g_L\Delta_T e^{\frac{V_m - V_T}{\Delta_T}} - I_{total} \tag{6}$$

where C is the membrane capacitance, g_L is the leak conductance, E_L is the resting potential, Δ_T is the slope factor (which determines the sharpness of the voltage threshold), and V_T is the threshold voltage. After emitting a spike, the membrane potential is reset to V_R and a refractory period, τ_R, is enforced during which neurons cannot emit another spike. Connections between neurons within and between populations were initialized probabilistically. Axonal conductance delays are implemented with delays of .5ms. Neural and synaptic parameter values were taken from the literature where possible and set empirically otherwise (**Supplementary Table T1**) [5–10].

The probability that each pyramidal cell projected to an AMPA or NMDA synapse on other pyramidal cells in the same population was 0.08, and that each inhibitory interneuron projected to a $GABA_A$ synapse on other interneurons in the same population was 0.1. Pyramidal cells of each population projected to AMPA or NMDA synapses on the inhibitory interneurons with probability 0.1, and each inhibitory interneuron projected to $GABA_A$ synapses on the

pyramidal cells with probability 0.2. The large-scale connectivity between populations was therefore fixed, but the connectivity between individual neurons in those populations was randomly generated according to the connection probability parameters, which were set empirically.

As detailed in the Methods, to simulate value-based decision making during the course of the task, the BAM was provided with inputs from the same Rescorla-Wagner rule that was part of the standard RL model used for modelling the behavioural experimental data. In addition to expected value inputs for each choice option, all neural populations in the BAM received noisy background input. Variations in the strength of the background input drive the stochasticity in the choice behaviour of the model [3]. To capture the range of stochasticity in human subjects without stimulation, we therefore needed to relate the firing rate of the background inputs to the BAM to the estimated *inverse temperature*, *β,* in the RL model used to analyse behaviour. In pilot simulations, we ran the BAM using a fixed learning rate and various levels of background noise. After fitting the behavioural output of the BAM with the standard RL model, we found that the estimated choice stochasticity (i.e. *inverse temperature*) without stimulation scaled linearly with the frequency of background noise. We therefore used linear regression to fit the firing rate of the background input, f_b, to the estimated *inverse temperature, β*:

$$f_b = \frac{161.08 - \beta}{0.17} Hz \qquad (7)$$

Using this equation (7) we were thus able to generate an instantiation of the BAM based on the variability in choice behaviour estimated from each human subject in the no stimulation control condition.

Alternative simulations

In addition to simulating the effect of anodal (depolarizing) stimulation we also simulated the effect of cathodal (hyperpolarizing) stimulation over vmPFC. Our main simulations were based on previous work [11], and so we tested how variation of the parameters of this work influenced model outcomes. First, we altered the stimulation parameters for interneurons in our model (Alternative 1-2), given that interneurons might be affected by a much smaller degree by polarizing currents, and uncertainty on how their morphology and orientation will affect polarization [11,12]. Second, we omitted polarization from pyramidal neurons (Alternative 3). This is in contrast with known physiology of polarizing currents [11,12] and served as an additional test for the specificity of our simulated membrane polarization effects on the BAM.

Current simulations

Complete methods for MRI-derived head models of EF distributions are detailed elsewhere[13]. Briefly, EF distributions in the brain were computed using individualized head models created from the T1-weighted MRI scans of a healthy individual. The head was segmented into compartments representing grey matter, white matter, cerebrospinal fluid, skull, scalp, eye region, muscle, air and blood vessels using a combination of tools from the FSL and Simpleware (Exeter, UK). The finite element (FE) mesh generated from the segmentation masks was exported to COMSOL Multiphysics 4.2 (Burlington, MA, USA) for computation of EFs.

Data analyses

Behavioural data of the human subjects were analysed using SPSS (Release 15.0.0, Chicago, USA), while behavioural data of virtual subjects and mean firing rates of the BAM were analysed using SciPy. Non-parametric tests (Wilcoxon test for comparing two repeated measures and Friedman test for comparing more than two repeated measures) were used when parametric test assumptions were violated. Effect size r on the Wilcoxon test was calculated as Z divided by the square root of the number of subjects.

Supplementary Results

Control Analysis 1: Termination of stimulation before task completion

Since tDCS was performed for 15 min, and started 5 min before task performance, stimulation was on average terminated about 3 minutes before the task was completed (mean task duration 13 minutes). We examined whether this might have affected our results by comparing choice accuracy of the whole dataset and a dataset excluding the last third of the trials (approximating the time the stimulation was present). We did not observe significantly different results (non-parametric tests for lateral depolarizing stimulation $p = .52$, medial depolarizing stimulation $p = .39$, and for control $p = .17$). We therefore conclude that the termination of the stimulation before task completion did not affect our results to a statistically measurable degree (see **Supplementary Figure 3**).

Control Analysis 2: Stimulation effects on choice accuracy on easy and hard trials, model and empirical data

As illustrated in **Figure 2**, baseline noise might bias selection of the option corresponding to the population with the initially higher firing rate, in particular when the two choice options have similar expected values (difficult trials). When exploring the stimulation effect on the choice accuracy, a somewhat more complicated pattern emerges, due to the fact that choice accuracy is also dependent on overall performance levels. Specifically, we examined the effect of stimulation on the accuracy of modelled as well as empirical choices for the 25% of trials with largest absolute difference in expected value between the two choice options (easy), and the 25% with the smallest absolute value difference (hard). We observe in both model as well as empirical data that medial frontal stimulation causes a bigger decrement in choice accuracy in the easy as opposed to the hard subset of trials (see **Supplementary Figure 4**). This effect is only significant in the model data, possibly due to noisier empirical as compared to model data (model data: effect size of correct choices in depolarizing stimulation relative to control (%) compared on easy versus hard trials: $Z = 3.0$, $p < .01$, empirical data: $p = .78$). Mechanistically, our model suggests that on difficult trials, the influence of value difference on choice is small compared to the influence of the background noise, and the performance is nearly at chance level. Depolarizing stimulation increases the pre-stimulus bias (**Figure 2**), but also pushes performance towards chance levels. Given performance levels are already close to chance (cf. **Supplementary Figure 4**), the potential effect of stimulation is limited. On easy trials, by contrast, the large expected value difference can on most trials overcome the background noise, but depolarizing stimulation amplifies the impact of the noise. This can cause incorrect decisions in some trials, leading to a larger decrement in choice accuracy in the easier subset of trials.

References

1. Bonaiuto, J. & Arbib, M. A. Modeling the BOLD correlates of competitive neural dynamics. *Neural Netw. Off. J. Int. Neural Netw. Soc.* **49,** 1–10 (2014).
2. Hunt, L. T. *et al.* Mechanisms underlying cortical activity during value-guided choice. *Nat. Neurosci.* **15,** 470–476 (2012).
3. Wang, X.-J. Probabilistic decision making by slow reverberation in cortical circuits. *Neuron* **36,** 955–968 (2002).
4. Wang, X.-J. Decision Making in Recurrent Neuronal Circuits. *Neuron* **60,** 215–234 (2008).
5. Hestrin, S., Sah, P. & Nicoll, R. A. Mechanisms generating the time course of dual component excitatory synaptic currents recorded in hippocampal slices. *Neuron* **5,** 247–253 (1990).
6. Jahr, C. E. & Stevens, C. F. A quantitative description of NMDA receptor-channel kinetic behavior. *J. Neurosci.* **10,** 1830–1837 (1990).
7. McCormick, D. A., Connors, B. W., Lighthall, J. W. & Prince, D. A. Comparative electrophysiology of pyramidal and sparsely spiny stellate neurons of the neocortex. *J. Neurophysiol.* **54,** 782–806 (1985).
8. Salin, P. A. & Prince, D. A. Spontaneous GABAA receptor-mediated inhibitory currents in adult rat somatosensory cortex. *J. Neurophysiol.* **75,** 1573–1588 (1996).
9. Spruston, N., Jonas, P. & Sakmann, B. Dendritic glutamate receptor channels in rat hippocampal CA3 and CA1 pyramidal neurons. *J. Physiol.* **482,** 325–352 (1995).
10. Xiang, Z., Huguenard, J. R. & Prince, D. A. GABAA receptor-mediated currents in interneurons and pyramidal cells of rat visual cortex. *J. Physiol.* **506,** 715–730 (1998).
11. Molaee-Ardekani, B. *et al.* Effects of transcranial Direct Current Stimulation (tDCS) on cortical activity: A computational modeling study. *Brain Stimulat.* **6,** 25–39 (2013).
12. Márquez-Ruiz, J. *et al.* Transcranial direct-current stimulation modulates synaptic mechanisms involved in associative learning in behaving rabbits. *Proc. Natl. Acad. Sci. U. S. A.* **109,** 6710–6715 (2012).
13. Datta, A. *et al.* Gyri-precise head model of transcranial direct current stimulation: Improved spatial focality using a ring electrode versus conventional rectangular pad. *Brain Stimulat.* **2,** 201–207.e1 (2009).

Supplementary Figure S1

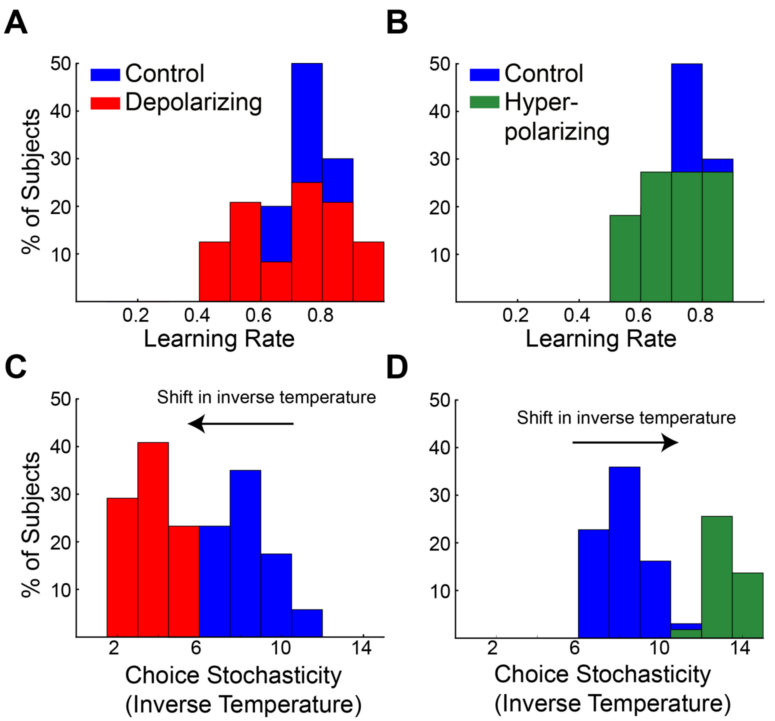

Supplementary Figure S2

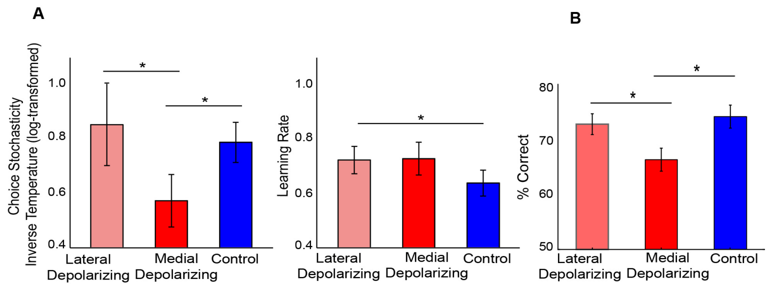

Supplementary Figure S3

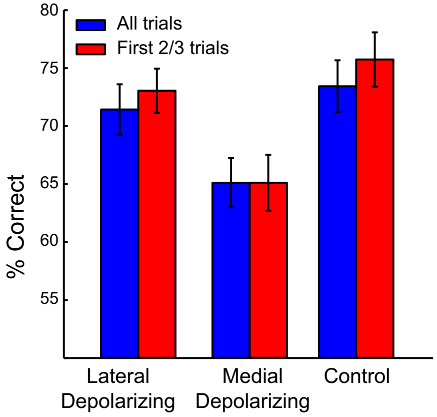

Supplementary Figure S4

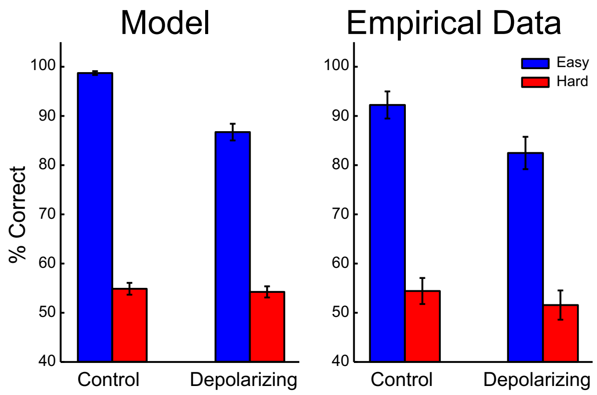

Supplementary Table 1

Parameter	Description	Value
$G_{AMPA(ext)}$	Maximum conductance of AMPA synapses from external inputs	2.1nS (pyramidal cells), 1.62nS (interneurons)
$G_{AMPA(rec)}$	Maximum conductance of AMPA synapses from recurrent inputs	0.05nS (pyramidal cells), 0.04nS (interneurons)
G_{NMDA}	Maximum conductance of NMDA synapses	0.165nS (pyramidal cells), 0.13nS (interneurons)
$G_{GABA\text{-}A}$	Maximum conductance of $GABA_A$ synapses	1.3nS (pyramidal cells), 1.0nS (interneurons)
τ_{AMPA}	Decay time constant of AMPA synaptic conductance	2ms
$\tau_{1\text{-}NMDA}$	Decay time constant of NMDA synaptic conductance	2ms
$\tau_{2\text{-}NMDA}$	Rise time constant of NMDA synaptic conductance	100ms
$\tau_{GABA\text{-}A}$	Decay time constant of $GABA_A$ synaptic conductance	5ms
$[Mg^{2+}]$	Extracellular magnesium concentration	1mM
E_{AMPA}	Reversal potential of AMPA-induced currents	0mV
E_{NMDA}	Reversal potential of NMDA-induced currents	0mV
$E_{GABA\text{-}A}$	Reversal potential of $GABA_A$-induced currents	-70mV
C	Membrane capacitance	0.5nF (pyramidal cells), 0.2nF (interneurons)
g_L	Leak conductance	25nS (pyramidal cells), 20nS (interneurons)
E_L	Resting potential	-70mV
Δ_T	Slope factor	3mV
V_T	Voltage threshold	-55mV
V_s	Spike threshold	-20mV
V_r	Voltage reset	-53mV
τ_r	Refractory period	2ms (pyramidal cells), 1ms (interneurons)

Supplementary Table T2:

Condition:	Depolar. Altern. 1	Depolarizing Altern. 2	Depolarizing Altern. 3	Hyperpolarizing Altern. 1	Hyperpolarizing Altern. 2	Hyperpolarizing Altern. 3
**Pyramidal neurons**	4pA	4pA	OFF	-4pA	-4pA	OFF
**Interneurons**	OFF	2pA	-2pA	OFF	-2pA	2pA
**Change in *Learning rate***						
**compared to no stimulation**	W=132.0, Z=-∞, p=1.0	W=109.0, Z=-∞, p=1.0	W=146.0, Z=-∞, p=1.0	W=153.0, Z=-∞, p=1.0	W=156.0, Z=-∞, p=1.0	W=144.0, Z=-∞, p=1.0
**compared to main stimulation condition**	W=147.0, Z=-∞, p=1.0	W=135.0, Z=-∞, p=1.0	W=161.0, Z=-∞, p=1.0	W=138.0, Z=-∞, p=1.0	W=145.0, Z=-∞, p=1.0	W=141.0, Z=-∞, p=1.0
**Change in choice stochasticity (*Inverse temperature)***						
**compared to no stimulation**	W=26.0, Z=2.93, P<.01, r = .59	W=39.0, Z=2.50, p = 0.01, r = .50	W=70.0, Z=1.34, p=0.18	W=7.0, Z=3.54, p< .01, r = .71	W=35.0, Z=2.63, p< .01, r = .53	W=36.0, Z=2.60, p< .01, r = .52
**compared to main stimulationcondition**	W=23.0, Z=3.03, p< .01, r=.60	W=0.0, Z=3.76, p< .01, r=.75	W=10.0, Z=3.45, p< .01, r = .71	W=62.0, Z=1.67, p=0.09, r=.33	W=68.0, Z=1.43, p=0.15	W=40.0, Z=2.46, p=0.01, r=.49
**Change in Choice Accuracy**						
**compared to no stimulation**	W=0.0, Z=3.69, p< .01, r=.74	W=34.5, Z=2.47, p=0.01, r=.49	W=29.5, Z=2.64, p< .01, r=.53	W=117.5, Z=-∞, p=1.0	W=160.5, Z=-∞, p=1.0	W=137.0, Z=-∞, p=1.0
**compared to main stimulation condition**	W=54.0, Z=1.92, p=0.05, r=.39	W=8.0, Z=3.51, p< .01, r=.70	W=9.0, Z=3.43, p< .01, r=.69	W=98.0, Z=-0.20, p=1.0	W=66.0, Z=1.51, p=0.13	W=73.0, Z=1.21, p=0.22
**Change in pyramidal prestimulus firing rate (difference chosen – unchosen)**						
**compared to no stimulation**	W=0.0, Z=3.75, p< .01, r=.75	W=20.0, Z=3.13, p < *.01*, r=.63	W=10.0, Z=3.45, p< .01, r=.69	W=3.0, Z=3.66, p< .01, r=.73	W=18.0, Z=3.19, p< .01, r=.64	W=23.0, Z=3.03, p< .01, r=.61
**compared to main stimulation condition**	W=3.0, Z=3.66, p< .01, r=.73	W=1.0, Z=3.73, p< .01, r=.75	W=0.0, Z=3.76, p< .01 , r=.75	W=96.0, Z=-0.04, p=1.0	W=52.0, Z=2.04, p=0.04, r=.41	W=15.0, Z=3.29, p< .01, r=.66
**Change in baseline interneuron firing rate**						
**compared to no stimulation**	W=0.0, Z=3.76, p < .01, r>.75	W=0.0, Z=3.76, p < .01, r>.75	W=109.0, Z=-∞, p=1.0	W=0.0, Z=3.76, p < .01, r>.75	W=0.0, Z=3.76, p < .01, r>.75	W=8.0, Z=3.51, p < .01, r>.70
**compared to**	W=109.0,	W=115.0,	W=0.0,	W=0.0,	W=0.0,	W=0.0,

**main stimulation condition**	Z=-∞, p=1.0	Z=-∞, p=1.0	Z=3.76,<br>p< .01,<br>r=.75	Z=3.756,<br>p < .01,<br>r>.70	Z=3.76,<br>p < .01,<br>r>.70	Z=3.76,<br>p < .01,<br>r>.70

Supplementary Figure Legends

Supplementary Figure 1: Simulated effects of neurostimulation on behavioural parameters

Distributions of estimated learning rates and choice stochasticity (inverse temperature) from the biophysical attractor model before and after depolarizing and hyperpolarizing stimulation. Neither stimulation condition had an effect on learning rate (A and B). Depolarizing stimulation increased choice stochasticity (decreased inverse temperature) (C), while hyperpolarizing stimulation decreased choice stochasticity (increased inverse temperature) (D).

Supplementary Figure 2: Effects of control stimulation over lateral PFC on behavioural parameters and choice accuracy

A) In line with model predictions, choice stochasticity was increased (and inverse temperature decreased) during medial depolarizing stimulation. This effect was not observed for lateral stimulation. No stimulation effect is present on learning rate.

B) Simulated as well as empirical data show a reduction in % correct choices during (medial) depolarizing stimulation. Correct choices are defined as the percentage of trials where the higher-valued option was chosen (expected value = reward magnitude * modelled reward probability).

Supplementary Figure 3: Effects of terminating stimulation before task completion

Mean choice accuracy on complete task (blue) and on task excluding the last third of trials (red, approximating the time the stimulation was present) did not differ for experimental conditions. Errorbars show +/-1 SE.

Supplementary Figure 4: Stimulation effects on choice accuracy on easy and hard trials, model and empirical data

Mean model-predicted and empirical choice accuracy for easy and hard trials (lowest and highest quartile in expected value difference between options). Errorbars show +/-1 SE.

Supplementary Table T1: Parameter values for the neural model used in the simulations.

Supplementary Table T2: Stimulation parameters used for alternative current simulations and resulting changes in physiological and behavioural model parameters

Note: Main stimulation condition refers to main depolarizing condition for depolarizing alternative simulations and to main hyperpolarizing condition for hyperpolarizing alternative simulations (as reported in Figures 2 through 4)
